# Assembly of a functional and responsive microstructure by heat bonding of DNA-grafted colloidal brick

**DOI:** 10.1038/s41598-017-09804-y

**Published:** 2017-08-22

**Authors:** Yuki Sakamoto, Shoichi Toyabe

**Affiliations:** Department of Applied Physics, Graduate School of Engineering, Tohoku University, Sendai, 980-8579 Japan

## Abstract

A micromachine constructed to possess various chemical and mechanical functions is one of the ultimate targets of technology. Conventional lithographic processes can be used to form complicated structures. However, they are basically limited to rigid and static structures with poor surface properties. Here, we demonstrate a novel method for assembling responsive and functional microstructures from diverse particles modified with DNA strands. The DNA strands are designed to form hairpins at room temperature and denature when heated. Structures are assembled through the simultaneous manipulation and heating of particles with “hot” optical tweezers, which incorporates the particles one by one. The flexible connection formed by DNA strands allows the responsive deformation of the structures with local controllability of the structural flexibility. We assembled a microscopic robot arm actuated by an external magnet, a hinge structure with a locally controlled connection flexibility and a three-dimensional double helix structure. The method is simple and can also be applied to build complex biological tissues from cells.

## Introduction

Diverse microrobots have been produced using lithographic methods for use in medical applications such as drug delivery systems^1–3^, including a flagellum-like helical structure actuated magnetically^4^, a cylinder actuated chemically, and a nanowire actuated acoustically^4–6^. These previous efforts focused on the swimming actuations needed to reach the target, such as cancer cells. The next challenge would be to implement functions to allow interaction with the target by, for example, mechanical or chemical actions. It is necessary to establish a methodology for assembling microscopic structures capable of responsive deformations and that possess a diverse and inhomogeneous functional surface.

DNA origami has been celebrated for its use in constructing submicron-sized structures by folding a long DNA scaffold strand with short staple strands into designed structures^7–12^. By using modified staple strands, the final structure can be readily imparted with chemical and physical properties. Rigid structure is an important factor for mechanical machines. Although DNA strands are basically flexible, a bundled structure can have a rigidity sufficient for working as mechanical device^13^. On the other hand, when we think of assembling a micron-sized machine, methods based on only DNA strands would be not the best choice. The dimension is basically limited by the DNA scaffold size to be around 100 nm. A technique to assemble larger micron-size structures by combining multiple DNA origami structures has been developed^14, 15^. However, it is still a challenge to construct a complex and rigid mechanical structure in micrometer scale.

An alternative approach is to use colloidal particles as building bricks to assemble structures^16–20^. Because colloidal particles are widely used in biotechnology applications, particles ranging from 10 nm to 10 *μ*m with a variety of chemically, physically, magnetically, and optically functional properties are commercially available. A strategy for constructing complex microrobots with colloidal particles would have great potential, as a structure could easily be given various functions by incorporating functionalized particles. This approach circumvents the dimension and rigidity problem inherent with DNA origami. Especially, modification of the particle surface with DNA strands provides a high controllability of the interactions between particles and has produced various colloidal crystals^16^. However, the colloidal complexes built with the conventional self-organization process are limited to geometric crystal structures.

## Experiments

**Figure 1:**
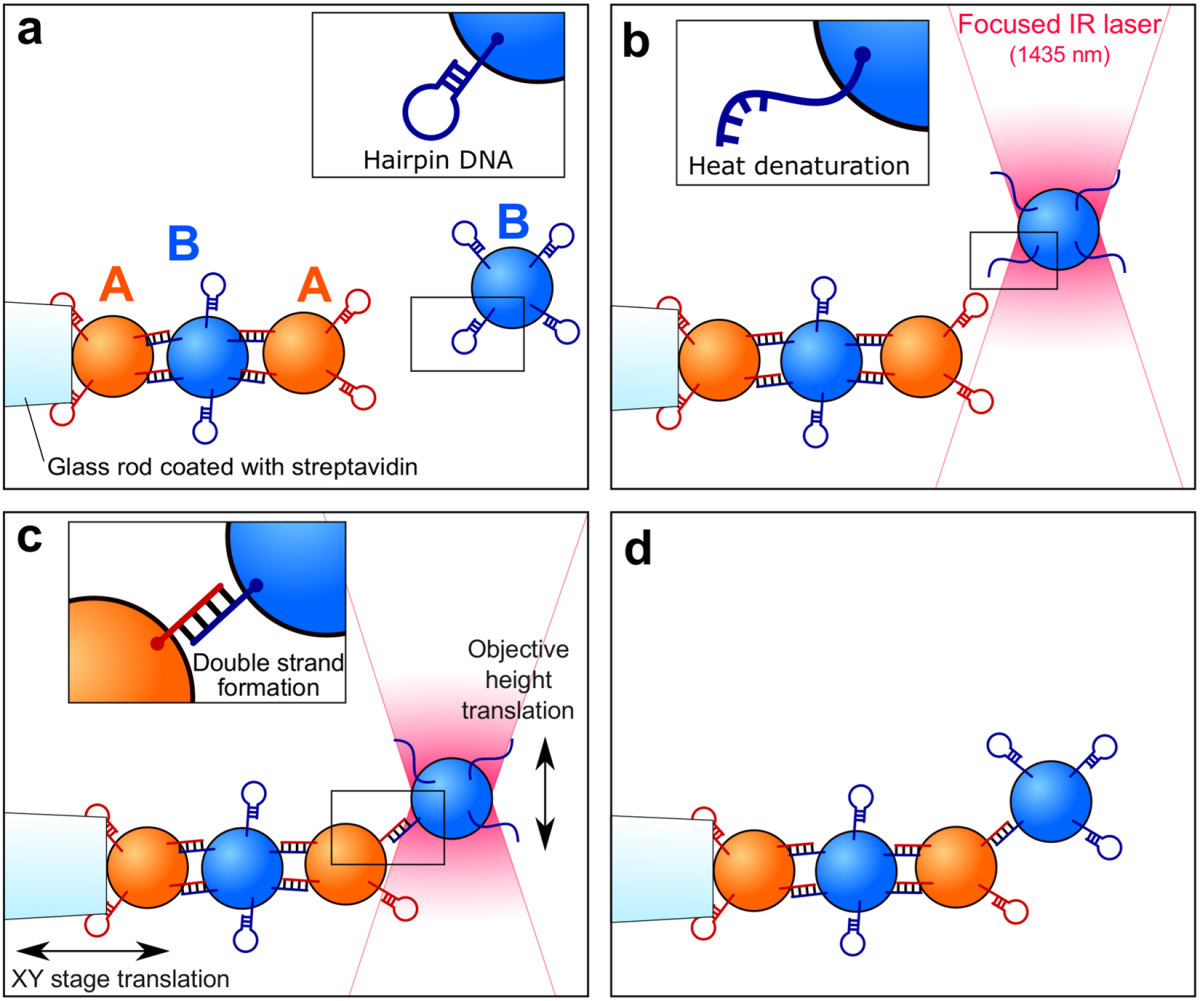
The assembly of microscopic structures by heat bonding. (**a**) Particles that serve as assembling bricks are suspended in a buffer. A complementary pair of hairpin DNA strands, A (red) and B (blue), are modified on the particles. (**b**) An infrared laser (1435 nm) focused under a microscope can trap the particle and manipulate it in three dimensions. Simultaneously, the same focused laser heats the water locally, causing the hairpin DNA strands on the particle surface to unfold. (**c**) The trapped particle is brought close to the structure by translating the chamber in the X and Y directions and the objective lens in the Z direction. The hairpin DNA strands on the side of the structure are also unfolded. The complementary DNA strands on the particles form double strands and connect the particles. (**d**) The particle remains connected to the structure even after the laser is turned off because of the hysteresis nature of the hairpin DNA strands (Fig. 2a).

In this letter, we demonstrate the construction of three-dimensional responsive microstructures by incorporating diverse particles one by one (Fig. 1). Our method relies on the heat bonding of colloidal particles modified with hairpin-loop DNA strands. The hysteresis nature of the hairpin folding/unfolding transition provides a hot glue mechanism to connect particles. The “hot optical tweezers” can simultaneously manipulate and heat these particles and assemble the structures. Diverse microscopic objects, including plastic particles, magnetic particles, quantum dots, and even biological cells, can be added to a structure at an arbitrary position once their surface is modified with the hairpin DNA strands.

**Figure 2:**
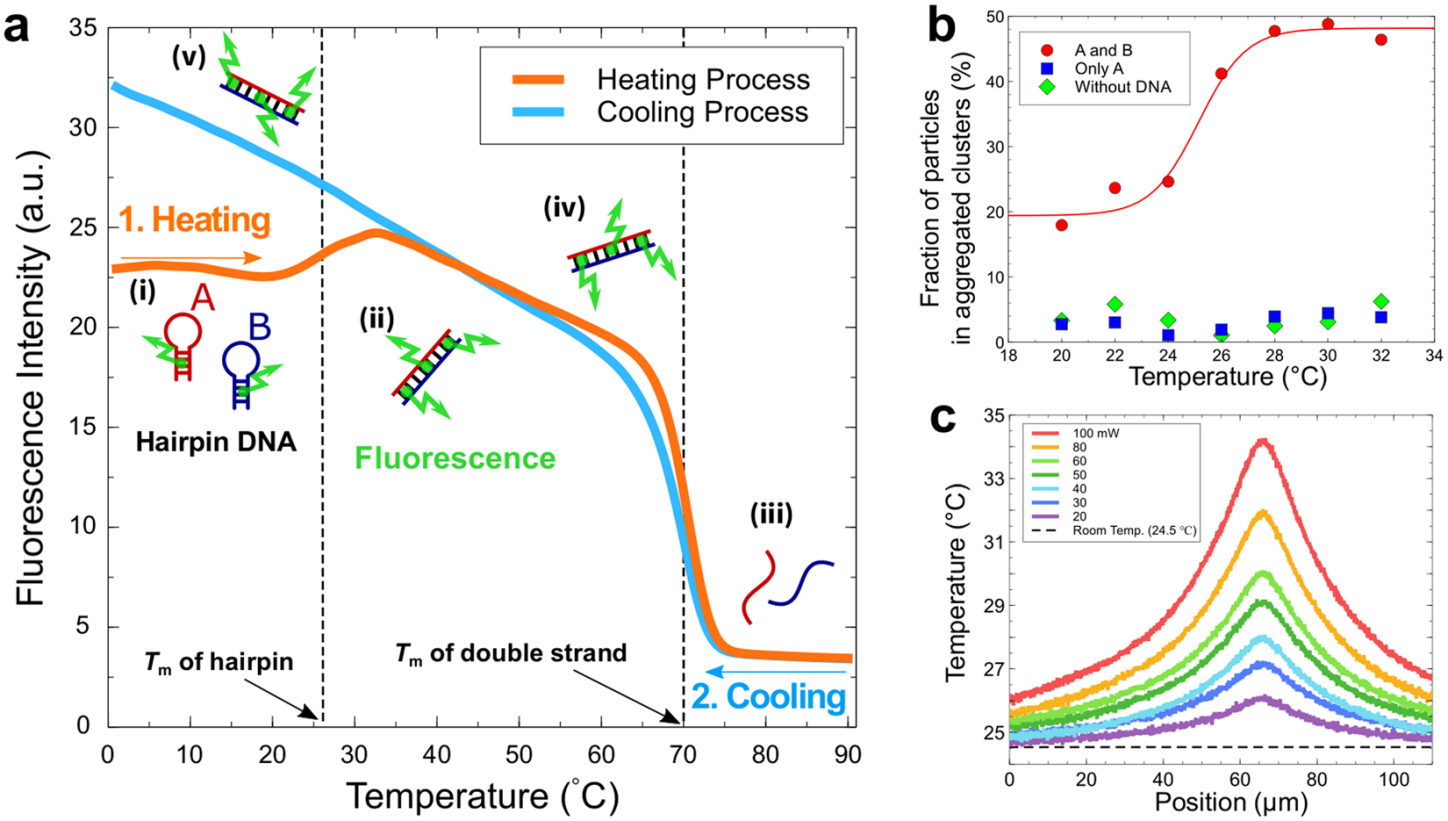
Temperature properties and temperature distribution. (**a**) Melting curve of the hairpin DNA strands. DNA strands A and B are suspended in the presence of EvaGreen, which is a fluorescent dye specific for double-stranded DNA. The fluorescent intensity indicates the amount of the double helices in the solution. The solution was heated from 0 °C to 90 °C and then cooled down to 0 °C. (i) A and B form hairpin-loop structures separately below *T*_m,hairpin_ = 26°C. (ii) When heated above *T*_m,hairpin_, the hairpin structures unfold, and A and B form double strands. (iii) At *T* > *T*_m,ds_ = 70 °C, the double strands denature, and fluorescence decreases. (iv) When cooled down below *T*_m,ds_, double strands form again. (v) When cooled down even below *T* < *T*_m,hairpin_, the double strands remain formed. (**b**) Aggregation test with 2 *μ*m DNA-colloidal particles. The particle suspension (2.0%v/v) was kept at 1 °C and then heated to the specified temperatures and incubated for 1 minute. After it was cooled down to 1 °C and diluted to 1/50, the particle aggregation was observed under a microscope. Some particles formed an aggregated cluster containing various number of particles. The number fractions of the particles in aggregated clusters are plotted. Because it was difficult to count the number of particles in a large cluster, we recorded the number of particles as four for the clusters containing four or more particles. This did not affect the qualitative result. We plotted the fraction of the particle numbers of the aggregated clusters in the suspension for both particles A and B (circles), only particles A (squares), and particles without DNA strands (diamonds). The solid curve is a fitting by a Tanh function. (**c**) Temperature distribution around the focused laser spot in the chamber filled with a buffer. At the laser focal point, the temperature was locally heated up to 9 °C according to the laser power. The temperature profile was well fitted by a Lorentzian curve (data not shown) as reported in the previous studies^25, 34^, validating our method of the local heating and the temperature measurement.

The hot optical tweezers use an infrared laser with a wavelength of 1435 nm, which is longer than that of conventional optical tweezers (1064 nm)^21^. The tweezers can simultaneously manipulate a particle and heat the water locally around the focal point because water has an absorption peak around this wavelength (Fig. 2c)^22, 23^. As a result of the heating, the hairpin DNA strands on the trapped particles are denatured (Fig. 1b). When a particle is placed close to complementary particles, the particles are connected by the double-strand formation (Fig. 1c). The connection is stable even after the laser is turned off because of the hysteresis character of the folding/unfolding transition of the DNA hairpin (Fig. 1d). More specifically, the hairpin state is a metastable state, and the double-stranded state is more stable. Once the DNA strands form a double strand, they do no longer form hairpins even after cooled down.

### Hairpin DNA strands

A complementary pair of hairpin-loop DNA strands (denoted as A and B) was designed so that (i) the melting temperature of the hairpin structure (*T*_m,hairpin_) is a few degrees higher than room temperature and (ii) the melting temperature of the double-stranded form of A and B (*T*_m,ds_) is much higher than room temperature (Fig. 2a). The hairpin structure is stable at room temperature but denatures when heated to *T* > *T*_m,hairpin_ and can hybridize to a DNA strand with a complementary sequence. These settings were inspired by the molecular beacon technique used to detect DNA and RNA strands with specific sequences^24^. The double strand is stable even after cooling down because of the hysteresis character and can act as a glue to connect particles.

The heat bonding of particles was tested by observing the particle aggregation under elevated temperatures. We prepared particles modified with A (particles A) and particles modified with B (particles B). If the particles are connected by DNA strands, the particles will aggregate. We observed aggregation only when a particle suspension containing both particles A and B was heated to above *T*_m,hairpin_ = 26 °C (Fig. 2b). Significant aggregation was not observed when the same solution was not heated or when the suspension contained only particles A or particles without DNA strands, demonstrating the high specificity of the heat bonding by the hairpin DNA strands.

### Local heating

The temperature increase induced by the hot optical tweezers was measured using the fluorescent ratio imaging of two fluorescent molecules, Rhodamine B and Rhodamine 101 (Sigma-Aldrich) (Fig. 2c)^25^. Their fluorescent intensities have a different temperature dependency. By measuring the fluorescence intensity ratio of the two molecules, we probed the spatial temperature variation without affecting the spatial concentration variation of the molecules due to thermophoresis. We found a localized temperature increase with a steep peak that had a width of about 20 *μ*m. A steady spatial pattern was formed in a time shorter than a video frame (16.7 ms). The temperature profile is determined by the size of the heated point, heat conduction in water, the chamber height, and the heat transfer from the chamber surface. The temperature at the peak increased with the laser intensity and was about 34 °C at the maximum under the measurement conditions used. This temperature increase was sufficiently above *T*_m,hairpin_ and enough to unfold the hairpin structures. In the following experiments, we used a laser power of 30 mW, which heats the solution to 27 °C at the peak.

### Structure assembly

**Figure 3:**
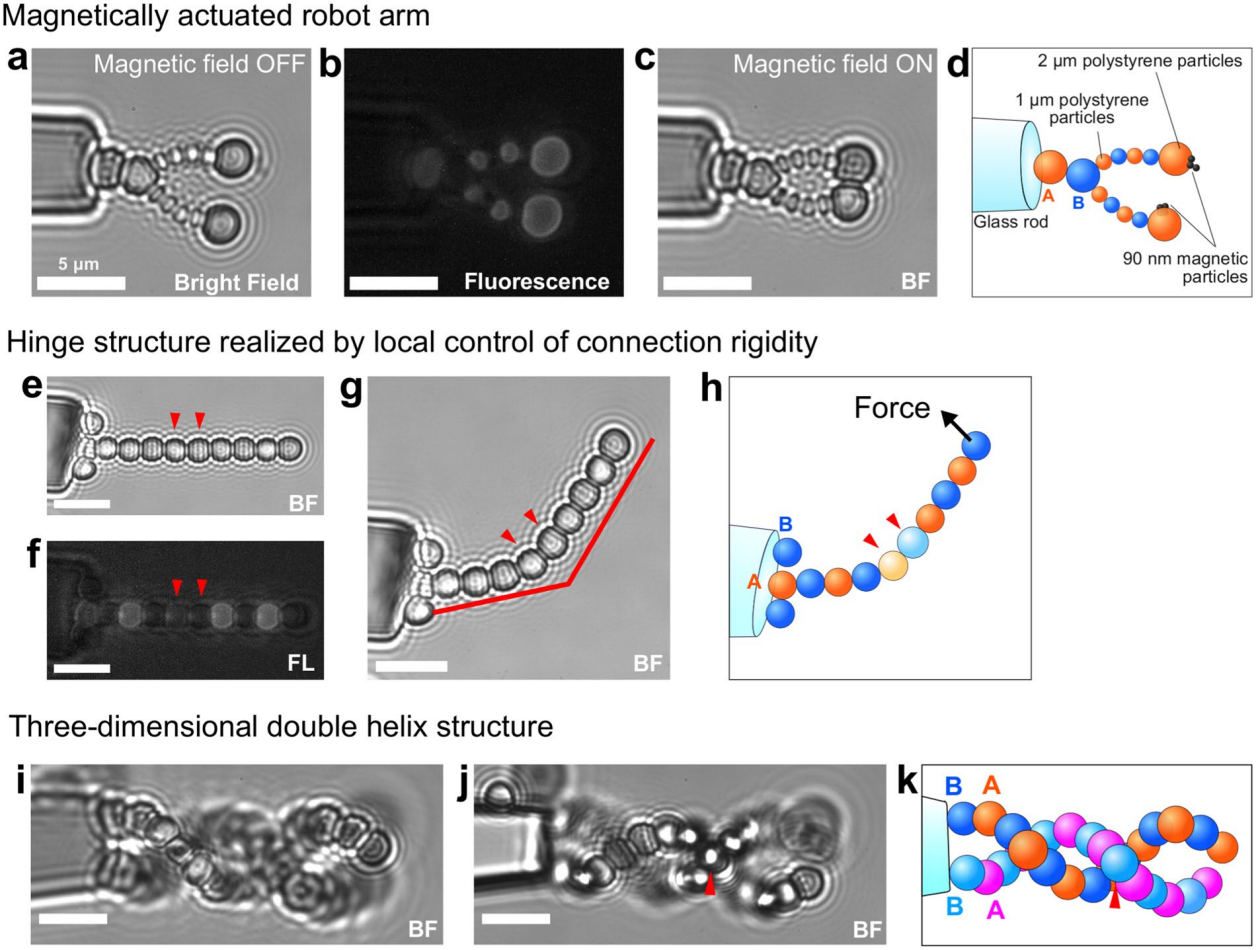
Microscopic structures assembled with the described method. (**a**) A microscopic robot arm actuated by an external magnet. The arm consists of 2-*μ*m and 1-*μ*m particles. Multiple 90-nm magnetic particles are fixed on the two tip particles. The arm opens and closes in response to an external magnet field. (**b**) Fluorescence image of the robot arm. Only strand A was modified with a HEX fluorescent dye on its 5′ end so that the A particles and B particles could be distinguished during the assembly process. (**c**) The robot arm closes when a permanent magnet (0.28 Tesla, diameter = 6 mm and height = 3 mm) is placed close to the chamber. (**d**) The schematic corresponding to (**c**). (**e**) Hinge structure. The primer A particle is supported by the B particles on the sides. The two particles indicated by red arrows have lower DNA densities than the other particles on the surface. The connections with these particles are more flexible and serve as a hinge. (**f**) Fluorescence image of the hinge structure. The fluorescence of the particles serving as the hinge is weak because of the low DNA density. (**g**) The structure bends at the hinge site when the tip particle is manipulated by the optical tweezers with a 40-mW laser. (**h**) The schematic corresponding to (**g**). (**i,j**) Three-dimensional double-helix structure. The two particle chains contact only at the point indicated by the red triangle. (**i** and **j**) show the images observed at different focal planes. (**k**) The schematic corresponding to (**i** and **j**). The two chains were drawn in different colors for ease of identification. Scale bars indicate 5 *μ*m. BF and FL denote bright field observation and fluorescent observation, respectively.

Using the method described above, we built a microscopic robot arm actuated by a magnetic field (Fig. 3a,d). The structure was assembled with different-sized polystyrene particles. Polystyrene particles with diameters of 1 *μ*m and 2 *μ*m were used in the arm part, and 2-*μ*m polystyrene particles coated with 90-nm magnetic particles were used in the arm tip. Particles A and B were alternately placed one by one. Only strand A was labeled with a HEX fluorescent dye on its 5′ end so that the A particles and B particles could be distinguished during the assembly process. This structure demonstrates that it is possible to incorporate diverse particles in arbitrary locations. When a permanent magnet was moved close to/away from the chamber, the arm opened and closed accordingly (Fig. 3a and c). This responsive movement is possible because of the flexible connection formed by the DNA strands.

The flexibility of the DNA connection is controllable by tuning the DNA concentration on the surface of the particles (see Supplementary Information). By reducing the DNA concentration on the particle surface, the bond between particles becomes weak. Figure 3e–h shows a hinge structure formed by incorporating fewer DNA strands into the particles, which in turn are placed at a specific site in the structure. We observed that the structure could bend at the site with these particles when a force was applied to the tip of the structure. This local flexibility control provides an additional degree of freedom to the mechanical function of the robot.

Finally, we assembled a three-dimensional double-helix structure (Fig. 3i–k). We attached particles one by one with a small shift in three dimensions by controlling the XY mechanical stage and the height of the objective lens, demonstrating that our method can be expanded to three-dimensional complicated structures. The construction of such a complex three-dimensional structure is limited when using conventional lithographic methods.

## Discussion

We demonstrated the assembly of a microstructure using DNA-grafted microbeads. With this method, it is possible to construct a machine with a variety of physical, chemical, optical, and magnetic functions by utilizing commercially available diverse colloidal particles. The method uses micron-sized particles and therefore can be used to construct a relatively large structure, which has yet to be achieved with DNA origami. On the other hand, the DNA strands provide a flexible connection between particles and make it possible to produce a responsive mechanical structure. One possible application of this method is to align biological cells to form a complex tissue structure^26–28^, and the modification of the cell surface with DNA strands has been established for such a purpose^29–31^. The heating causes a temperature increase of only a few degrees and can be applied to biological cells. Preparation of particles with localized DNA patches on the surface has been reported^20, 32^. The use of patchy DNA particles in our method would add more degrees of freedom for making complex structures.

Figure 2b demonstrates that the hairpin strands suppress the particle aggregation. In principle, by decreasing the particle concentrations sufficiently, we can suppress the aggregation and build structures even with DNA strands lacking hairpin structures. However, a structure assembly with such dilute particles is quite inefficient. Preparation of dense particle solution without aggregation is a key for the efficient assembly. It would be a future work to examine the relation between the hairpin stability and the particle aggregation.

## Methods

### DNA modification on particle surface

We used carboxylated particles (1 *μ*m from Molecular probes (1.1 ± 0.1 *μ*m), 2 *μ*m from Bangs Lab (2.19 ± 0.2 *μ*m), 90 nm magnetic particle (95 ± 5 nm) from Thermo Fisher Scientific). Sequences of DNA strands A and B are 5′-TGC CAT AAC AAA CGC TCC GAC ACA GAA TGG CT-3′ and 5′-TGC CAT TCT GTG TCG GAG CGT TTG TTA TGG CT-3′, respectively (Fig. S1). The DNA strands with amino groups on their 3′ ends (Eurofins Genomics) were modified on the surface of the particles as follows. 2.0%(v/v) of carboxylated particles suspended in 50 mM 2-Morpholinoethanesulfonic acid (MES, pH5.2) buffer were mixed with 3 *μ*M the DNA strands and 10 mg/mL N-(3-Dimethylaminopropyl)-N’ -ethylcarbodiimide hydrochloride (EDAC, Sigma-Aldrich, MO) and incubated for 180 minutes at room temperature. EDAC dehydrates the carboxyl group and the amino group to produce an amide bond and binds the DNA strand on the particle surface strongly by a covalent bond. The modified particles were resuspended in Tris buffer(10 mM Tris-HCl, 60 mM NaCl, pH 8.0). It was not possible to trap the 90-nm magnetic particles by the optical tweezers. Instead, we mixed the 90-nm magnetic particles modified with strands B and the 2-*μ*m particles modified with strands A and kept the mixture at 72 °C for 10 minutes to form particle complexes. 72 °C is slightly higher than the melting temperature of the double-stranded form of A and B and the temperature was experimentally determined as a temperature to form the particle complexes. The particle complexes were used to incorporate the magnetic particles into structures. See Fig. S2 and Table S1 for details.

### DNA melting curve

The melting curves of 1.0 uM hairpin DNA strands diluted in Tris buffer (10 mM Tris-HCl, 60 mM NaCl, pH 8.0) were obtained with a realtime PCR cycler (Biorad, CA) using a double-strand-specific 1x EvaGreen fluorescent dye (Biotium, CA).

### Aggregation test

2.0%(v/v) of 2.0-*μ*m particles were suspended in Tris buffer (10 mM Tris-HCl, 60 mM NaCl, pH 8.0). The particle suspension (2.0%v/v) was kept at 1 °C and then heated to the specified temperature and incubated for 1 minute. After the suspension was cooled down to 1 °C and diluted to 1/50, the particle aggregation was observed under a microscope. See Fig. S3 for details.

### Microscope

An infrared laser (1435 nm, QPhotonics, MI) was introduced to an inverted microscope (Olympus, Japan) with a 100x objective lens (NA 1.4, Olympus) and XYZ manual stage. The images were captured with a CMOS camera (Basler, Germany). We used benzyl alcohol (Sigma-Aldrich, MO) as the immersion oil to improve trapping ability^33^. For the temperature measurement, the fluorescence intensities of 50 mg/L Rhodamine B and 30 mg/L Rhodamine 101 (Sigma-Aldrich, MO) were measured by a CMOS camera and the intensity ratio of these two dyes (Fig. S5) are compared with the calibration curve (Fig. S4) obtained by a realtime PCR cycler (BioRad, CA)^25^.

## Electronic supplementary material

Supplemantry Information

Supplementary Movie 1

Supplementary Movie 2

Supplementary Movie 3

Supplementary Movie 4
