# Supplementary material for: Assembly of a functional and responsive microstructure by heat bonding of DNA-grafted colloidal brick
Source: Sci Rep. 2017 Aug 22;7:9104. doi: 10.1038/s41598-017-09804-y (PMC5567359; doi:10.1038/s41598-017-09804-y)
Supplement: Supplementary file 1 — Supplemantry Information [file 41598_2017_9804_MOESM1_ESM.pdf]

Supplementary Information

Assembly of a functional and responsive microstructure by heat bonding of DNA-grafted colloidal brick

Yuki Sakamoto and Shoichi Toyabe*

Department of Applied Physics, Graduate School of Engineering, Tohoku University, Sendai 980-8579, Japan

E-mail: toyabe@tohoku.ac.jp

S1. Oligonucleotide Sequences and Modifiers

DNA strands with the following sequences (Eurofins Genomics) were modified on particle surfaces.

Strand A : [HEX] 5′ - TGCCATAACAAACGCTCCGACACAGAATGGCT - 3′ [AmC7]
Strand B : 5′ -TGCCATTCTGTGTCGGAGCGTTTGTTATGGCT - 3′ [AmC7]

[HEX] : Hexachloro-Fluorescein, [AmC7] : 3′-Amino-Modifier-C7-CPG

These DNA strands are designed to form hairpin-loop forms at room temperature (24.5 °C) (Fig. S1). Five nucleotides at both ends of each strand are self-complementary and form a stem structure.

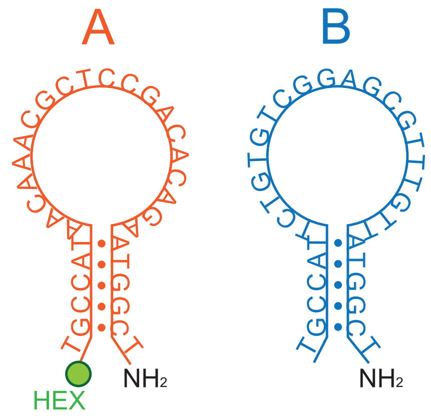

Figure S1: **DNA strand A and B.** These strands form hairpin structures at room temperature.

S2. DNA Strands Density on a Particle Surface

DNA strands density on a modified particle surface was estimated by measuring the residual fluorescence intensity of the modifying solution after the modification (Fig. S2). A calibration curve was obtained by measuring the fluorescent intensities of DNA strand A solution in a buffer containing 10 mg/mL of EDAC and 50 mM MES buffer (pH 5.20) with a realtime PCR cycler (BioRad) (Fig. S2).

After the modifications of the particle surface with 0.5, 1.0, or 3.0 μM of DNA strands A, we centrifuged the particle solutions and measured the fluorescence intensities of supernatant with the realtime PCR cycler. We compared them with the calibration curve to estimate the DNA density on the particle surface. The result was summarized in Table S1. This shows that most DNA strands in the modification buffer were modified on the particle surface in the measured range of the DNA concentration.

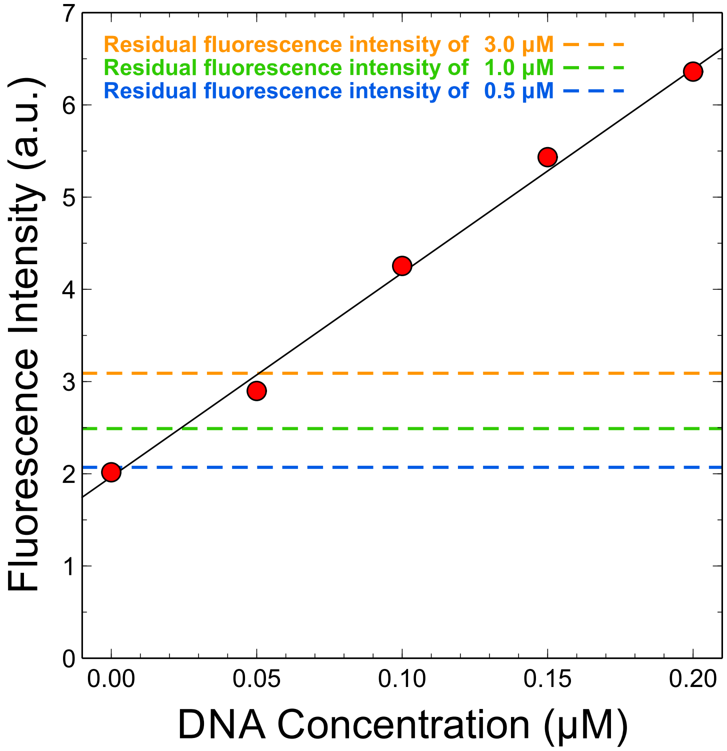

Figure S2: **Calibration curve for the relation between the DNA concentration and fluorescent intensity.** The fluorescent intensity of strand A, which has HEX fluorescent residue at the end, at various strand concentrations is plotted. The background level measured in a solution without the strand was 2.0. We obtained a calibration curve $y = 22x + 2.0$. The lines indicate the residual fluorescent intensity after the modification of particles by DNA.

Table S1: **Surface DNA strands density on 2-μm particles.** From the residual fluorescent intensity after the modification, the residual DNA concentration was obtained by using the calibration curve (Fig. S2). The amount of the strands modified on the particle was estimated by subtracting the initial strand concentration by the residual DNA concentration after the modification.

Initial strands [μM]	Residual FL intensity [a.u.]	Strands modified [μM]	Strands modified [/particle]	Distance between strands [nm]
3.0	3.09	2.95	4.9×10^5	2.5
1.0	2.49	0.98	1.6×10^5	7.4
0.5	2.07	0.49	8.2×10^5	15

S3. Particles Aggregation Test

We used 2-μm particles A and B modified with 3.0 uM DNA strands. First, 2.0%(v/v) of modified particles were suspended in a buffer (10 mM Tris-HCl, 60 mM NaCl, pH 8.0). The particle suspension (2.0%v/v) was kept at 1 °C and then heated to the specified temperature and kept for 1 minute. After cooling down to 1 °C and diluting to 1/50, we observed the particle aggregation under a microscope. We observed clusters with various sizes in the suspension containing both particles A and B, especially when they were heated to a temperature higher than 26 °C. Fraction of aggregated particles was around 50% at temperature higher than 28 °C. We did not observe a remarkable aggregation in a suspension containing only particles A nor only particles without DNA modifications.

S4. Temperature Distribution

Temperature distribution around the focused laser spot in the chamber was measured with the fluorescent ratio imaging of two fluorescent dyes,[1] Rhodamine B (Sigma-Aldrich, RhB) and Rhodamine 101 (Sigma-Aldrich, Rh101). These two dyes have different temperature dependencies. We used 50 mg/mL RhoB and 30 mg/mL Rho101 in a Tris buffer (10 mM Tris-HCl, 60 mM NaCl, pH 8.0).

Figure S4a shows the fluorescent intensities of the dyes at various temperature measured

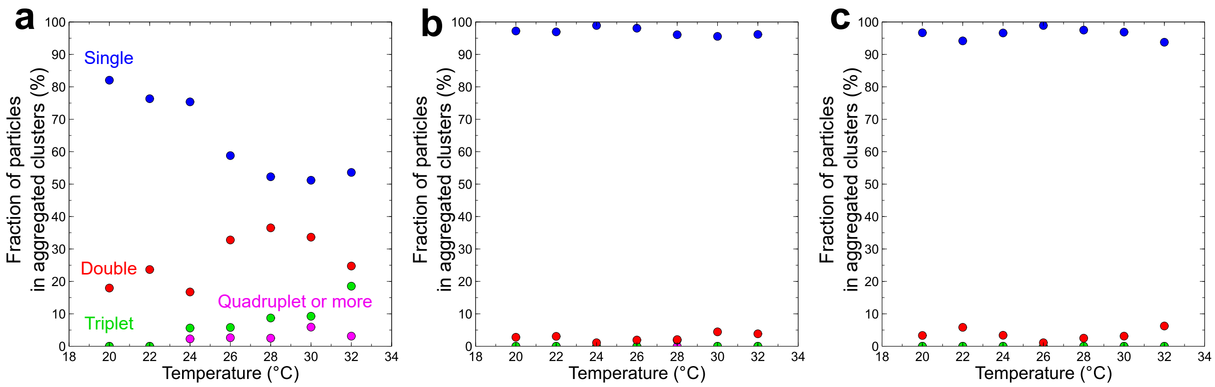

Figure S3: **Fractions of particles in aggregated clusters a,** Particles A and B. At 26 °C, about 40% of particles formed aggregated clusters. **b,** Particles A. Most of particles were kept separated as single particles, and small fraction of particles formed double-particle clusters. **c,** Particles without DNA.

by a real-time PCR cycler (BioRad). The fluorescence ratio (Fig. S4b) normalized at room temperature (24.5°C) was fitted by a logarithm curve $y = -35.2 \ln x + 24.5$. We obtained the spatial temperature distribution generated by the laser irradiation under a microscope by measuring the spatial fluorescence distribution in a chamber filled with RhoB or Rho101 (Fig. 2c in the main text).

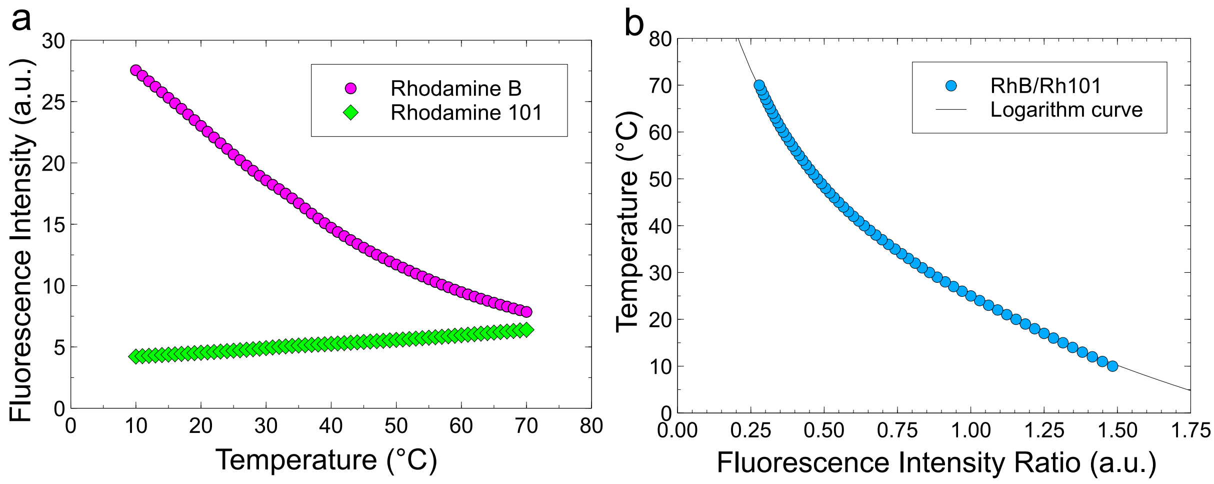

Figure S4: **Temperature dependency of fluorescent intensities of Rhodamine B and 101. a,** Fluorescence intensities of Rhodamine B and Rhodamine 101 at each temperature. Each has a different temperature dependence. **b,** The relation between the temperature and the fluorescence intensity ratio of Rhodamine B to Rhodamine 101. The ratio normalized at room temperature (24.5°C) was well fitted by a logarithm curve $y = -35.2 \ln x + 24.5$.

References

(1) Cordero, M. L.; Verneuil, E.; Gallaire, F.; Baroud, C. N. Time-resolved temperature rise in a thin liquid film due to laser absorption. *Phys. Rev. E* **2009**, *79*, 011201.

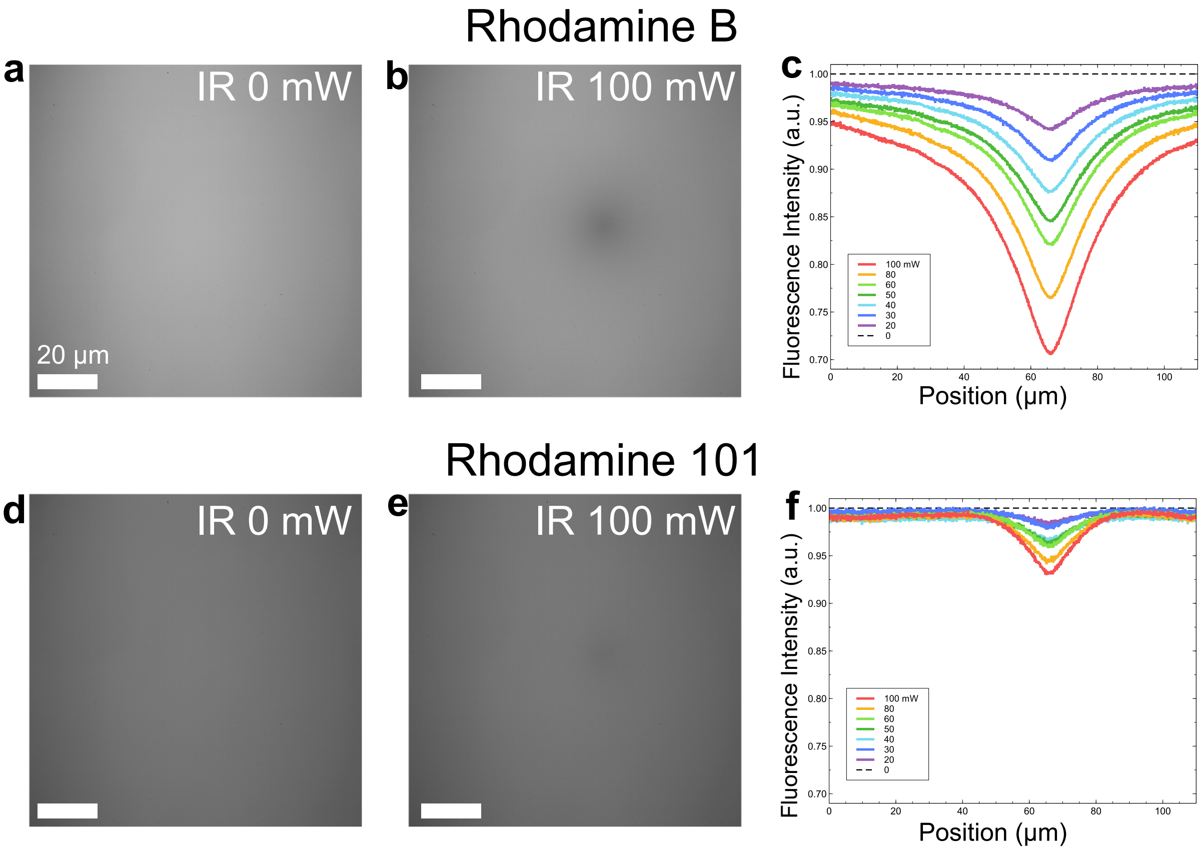

Figure S5: **Fluorescence distributions under IR irradiation.** 0 mW (**a**, **d**) or 100 mW (**b**, **e**) of IR is irradiated in the chambers filled with the solution of rhodamine B (**a**, **b**) or rhodamine 101 (**d**, **e**). Fluorescence around the focal point decreased. **c** and **f** show the normalized fluorescence distribution at various IR power. Scale bars are 20 μm.
